# The Cure of Tetanus with the Antitoxin Obtained from the Serum of an Immune Animal

**Published:** 1892-11

**Authors:** 


### The Cure of Tetanus with the Antitoxin Obtained from the Serum of an Immune Animal.

Casal (*Centralblatt f. Bacteriol. u. Parasitenk.*, xii, 2 u. 3, p. 56) has reported the case of a woman, twenty-two years old, who seven days after having received a lacerated wound of the right foot, and walking a considerable distance over damp ground with unprotected feet, presented manifestations of developing tetanus. For a week, under ordinary treatment, the symptoms progressively increased in intensity. Specific treatment was now proposed and assented to. Tetanus-bacilli were found in the pus from the wound on the foot. Six injections of the antitoxin prepared from the blood of a dog immune to tetanus were made at intervals of twelve hours ; the first five contained twenty-five centigrammes, and the sixth fifteen centigrammes. Improvement soon set in, and was progressive to perfect recovery.—*Med. News.*

